# 4-[(1,3-Dioxo-2,3-dihydro-1*H*-benzo[*de*]isoquinolin-2-yl)meth­yl]-*N*′-[(*E*)-4-nitro­benzyl­idene]benzene­sulfono­hydrazide dimethyl sulfoxide monosolvate

**DOI:** 10.1107/S1600536811004697

**Published:** 2011-02-12

**Authors:** Adailton J. Bortoluzzi, Everton B. Policarpi, Cristiano Mora, Kely N. Oliveira, Ricardo J. Nunes

**Affiliations:** a Departamento de Química, Universidade Federal de Santa Catarina, 88040-900 Florianópolis, Brazil

## Abstract

The mol­ecular structure of the title compound, C_26_H_18_N_4_O_6_S·C_2_H_6_OS, shows an *E* conformation of the hydrazone double bond. The presence of a methyl­ene group between the benzo[*de*]isoquinoline and benzene­sulfonyl moieties allows the 4-nitro­phenyl ring and the benzo[*de*]isoquinoline system to be parallel with respect to each other, so that the mol­ecule adopts a U-shaped spatial conformation. The dihedral angle between mean planes of these aromatic groups is 4.4 (1)°. This special arrangement enables neighboring mol­ecules to be inter­calated, forming slipped π–π inter­actions [centroid–centroid distance = 3.535 (2) Å] between the 4-nitro­phenyl and benzo[*de*]isoquinoline groups and point-to-face C—H⋯π inter­actions between the benzo[*de*]isoquinoline and benzene­sulfonyl aromatic systems. In addition, the crystal packing also features an inter­molecular N—H⋯O inter­action involving the amine group and the dimethyl sulfoxide solvent mol­ecule.

## Related literature

For the therapeutic properties of sulfonyl­hydrazones, see: Rollas *et al.* (2002 ▶); Frlan *et al.* (2008 ▶); Lima *et al.* (1999 ▶); Sondhi *et al.* (2006 ▶) and for their biological activity, see: Kendall *et al.* (2007 ▶); Sadek *et al.* (2008 ▶). For the anti­cancer activity of naphthalimides, see: Braña & Ramos (2001 ▶); Braña *et al.* (2001 ▶); Suárez & Sánchez (1992 ▶); Ingrassia *et al.* (2009 ▶); Wu *et al.* (2009 ▶); Norton *et al.* (2008 ▶). For the therapeutic properties of cyclic imides, see: Cechinel Filho *et al.* (2003 ▶); Walter *et al.* (2002 ▶). For background to this study, see: Silva *et al.* (2006 ▶); Oliveira & Nunes (2006 ▶).
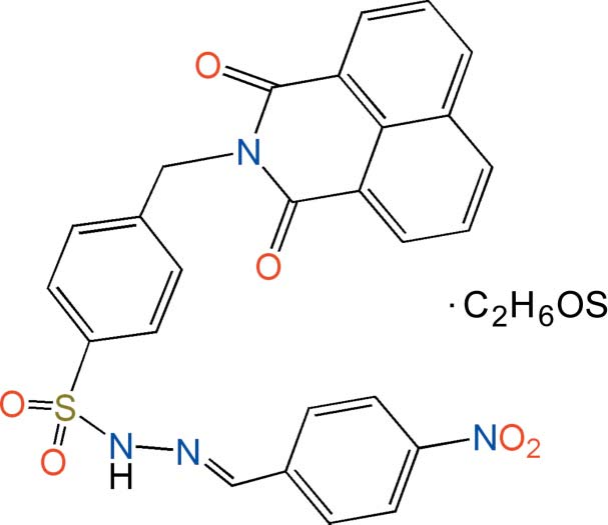


## Experimental

### 

#### Crystal data

C_26_H_18_N_4_O_6_S·C_2_H_6_OS
*M* *_r_* = 592.63
Triclinic, 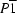

*a* = 9.152 (1) Å
*b* = 11.971 (1) Å
*c* = 13.910 (1) Å
α = 107.268 (7)°
β = 101.789 (7)°
γ = 96.319 (8)°
*V* = 1400.6 (2) Å^3^
*Z* = 2
Mo *K*α radiation
μ = 0.24 mm^−1^
*T* = 293 K
0.50 × 0.16 × 0.13 mm

#### Data collection

Enraf–Nonius CAD-4 diffractometer
5055 measured reflections
4737 independent reflections
3075 reflections with *I* > 2σ(*I*)
*R*_int_ = 0.017
3 standard reflections every 200 reflections  intensity decay: 1%

#### Refinement

*R*[*F* ^2^ > 2σ(*F* ^2^)] = 0.052
*wR*(*F* ^2^) = 0.163
*S* = 1.04
4737 reflections
371 parameters
H-atom parameters constrained
Δρ_max_ = 0.73 e Å^−3^
Δρ_min_ = −0.43 e Å^−3^

### 

Data collection: *CAD-4 Software* (Enraf–Nonius, 1989 ▶); cell refinement: *CAD-4 Software*; data reduction: *HELENA* (Spek, 1996 ▶); program(s) used to solve structure: *SIR97* (Altomare *et al.*, 1999 ▶); program(s) used to refine structure: *SHELXL97* (Sheldrick, 2008 ▶); molecular graphics: *PLATON* (Spek, 2009 ▶) and *Mercury* (Macrae *et al.*, 2006 ▶); software used to prepare material for publication: *SHELXL97*.

## Supplementary Material

Crystal structure: contains datablocks global, I. DOI: 10.1107/S1600536811004697/ff2001sup1.cif

Structure factors: contains datablocks I. DOI: 10.1107/S1600536811004697/ff2001Isup2.hkl

Additional supplementary materials:  crystallographic information; 3D view; checkCIF report

## Figures and Tables

**Table 1:** Hydrogen-bond geometry (Å, °) *Cg* is the centroid of the *p*-nitro­phenyl (C22–C27) ring.

*D*—H⋯*A*	*D*—H	H⋯*A*	*D*⋯*A*	*D*—H⋯*A*
N2—H2*N*⋯O1*S*	0.80	1.99	2.764 (4)	163
C8—H8⋯*Cg*^i^	0.93	2.90	3.799 (6)	162

Symmetry code: (i) 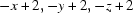.

**Fig. 1.**
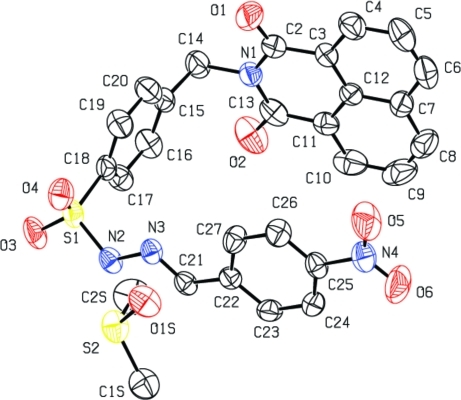
The molecular structure of the title compound with the labelling scheme. Displacement ellipsoids are shown at the 40% probability level.

**Fig. 2.**
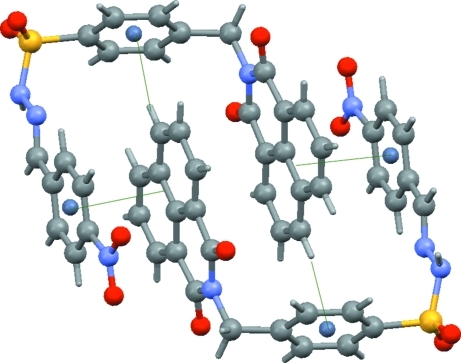
Intermolecular interactions observed in (I).

## supplementary crystallographic information

### Comment

Sulfonyl-hydrazones are known for their therapeutic properties, such as, antimicrobial (Rollas *et al.* 2002, Frlan *et al.* 2008), analgesic (Lima *et al.* 1999, Sondhi *et al.* 2006), *etc*. Sulfonyl-hydrazones was selective inhibitor of p110α, a phosphoinositide-3-kinase that is over-expressed in 30% of tumors (Kendall *et al.* 2007). In innovative study, the sulfonyl-hydrazones were reported by enhance myocardial repair by stem cells by activating cardiac differentiation in human mobilized peripheral blood mononuclear cells (*M*-PBMCs) (Sadek *et al.* 2008).

Cyclic imides have also many therapeutic properties including antibacterial, antitumor, diuretic and antiviral (Cechinel Filho *et al.* 2003). In a previous publication, we reported the synthesis of imidobenzenesulfonyl compounds that showed promising analgesic profiles in the acetic acid- induced mice writhing test. The mechanism of action occurred possibly due to additional non-covalent interactions with the COX active site (Walter *et al.* 2002). The naphthalimides, in special, are known of their high DNA-binding ability and consequently many of them have anticancer property (Braña & Ramos, 2001); Braña *et al.* 2001). Mitonafide and Amonafide are classical examples of naphthalimides derivatives with antitumoral activity (Suárez *et al.* 1992). Recently, many similar compounds have been showed activity against different cancer cell lines (Ingrassia *et al.* 2009, Wu *et al.* 2009, Norton *et al.* 2008).

The title compound (I) (Scheme 1) was synthesized as a part of our work to investigate the antitumoral activity of the sulfonyl-hydrazones cyclic imides derivatives (Oliveira *et al.* 2006, Silva *et al.* 2006).

The molecular structure of the title compound (Fig. 1) shows E conformation on the hydrazone double bond, which is evidenced by the torsion angle S1–N2–N3–C21 of 165.8 (2)°. The presence of a methylene group among benzo[*de*]isoquinoline and benzenesulfonyl moieties allows *p*-nitrophenyl ring and benzo[*de*]isoquinoline system to be parallel with respect to each other, so that the molecule adopts an U-shaped spatial conformation. The dihedral angle between mean planes of these planar groups is 4.4 (1)°. This special arrangement enables the neighboring molecule be intercalated by a center of symmetry, forming pairs of molecules in a centrosymmetric structure (Fig. 2). Slipped π–π interaction between *p*-nitrophenyl and benzo[*de*]isoquinoline, with centroid–C12 distance of 3.589 Å, and point-to-face C–H···π interaction between benzenesulfonyl and benzo[*de*]isoquinoline aromatic systems, with centroid-H8 distance of 2.903 Å, are observed. In addition, crystal packing also shows an intermolecular N2–H···O1S interaction involving amine group and DMSO solvate.

### Experimental

4-nitrobenzaldehyde (79 mg, 0.52 mmol) was added in a mixture of 4-[(1,3-dioxo-1*H*-benzo[*de*]isoquinolin-2(3*H*)-yl)methyl] benzenesulfonohydrazide (200 mg, 0.52 mmol) in ethanol (10 ml), with a drop of hydrochloric acid as catalyst, as described for similar compounds (Silva *et al.* 2006, Oliveira *et al.* 2006). The reaction was carried out by stirring at room temperature for one hour. The solid was filtered off with suction. The crystal used for data collection was obtained by dissolving 30 mg of (I) in 10 ml of dimethylsulfoxide and by slow evaporation of the solvent.

### Refinement

All non-H atoms were refined with anisotropic displacement parameters. H atoms were placed at their idealized positions with distances of 0.93 and 0.97 Å and *U*_eq_ fixed at 1.2 times *U*_iso_ of the preceding atom for C–H_Ar_ and C–H_2_, respectively and  at 1.5 times *U*_iso_ of the preceding atom for C–H_3_. The H atom of the amino group was found from Fourier map and treated with riding model and its *U*_eq_ was refined freely.

### Crystal data

**Table:**

C_26_H_18_N_4_O_6_S·C_2_H_6_OS	*Z* = 2
*M**_r_* = 592.63	*F*(000) = 616
Triclinic, *P*1	*D*_x_ = 1.405 Mg m^−^^3^
Hall symbol: -P 1	Mo *K*α radiation, λ = 0.71073 Å
*a* = 9.152 (1) Å	Cell parameters from 25 reflections
*b* = 11.971 (1) Å	θ = 8.5–13.4°
*c* = 13.910 (1) Å	µ = 0.24 mm^−^^1^
α = 107.268 (7)°	*T* = 293 K
β = 101.789 (7)°	Prismatic, colourless
γ = 96.319 (8)°	0.50 × 0.16 × 0.13 mm
*V* = 1400.6 (2) Å^3^	

### Data collection

**Table:**

Enraf–Nonius CAD-4 diffractometer	*R*_int_ = 0.017
Radiation source: fine-focus sealed tube	θ_max_ = 25.1°, θ_min_ = 2.3°
graphite	*h* = 0→10
ω–2θ scans	*k* = −14→14
5055 measured reflections	*l* = −16→16
4737 independent reflections	3 standard reflections every 200 reflections
3075 reflections with *I* > 2σ(*I*)	intensity decay: 1%

### Refinement

**Table:**

Refinement on *F*^2^	Primary atom site location: structure-invariant direct methods
Least-squares matrix: full	Secondary atom site location: difference Fourier map
*R*[*F*^2^ > 2σ(*F*^2^)] = 0.052	Hydrogen site location: inferred from neighbouring sites
*wR*(*F*^2^) = 0.163	H-atom parameters constrained
*S* = 1.04	*w* = 1/[σ^2^(*F*_o_^2^) + (0.0792*P*)^2^ + 0.613*P*] where *P* = (*F*_o_^2^ + 2*F*_c_^2^)/3
4737 reflections	(Δ/σ)_max_ < 0.001
371 parameters	Δρ_max_ = 0.73 e Å^−^^3^
0 restraints	Δρ_min_ = −0.43 e Å^−^^3^

### Fractional atomic coordinates and isotropic or equivalent isotropic displacement parameters (Å^2^)

**Table:**

	*x*	*y*	*z*	*U*_iso_*/*U*_eq_	
S1	0.34031 (11)	0.73620 (8)	0.53120 (6)	0.0597 (3)	
N1	0.8354 (3)	1.1992 (2)	0.8805 (2)	0.0571 (7)	
N2	0.4477 (3)	0.6426 (2)	0.55446 (19)	0.0540 (7)	
H2N	0.5170	0.6398	0.5273	0.058 (12)*	
N3	0.4776 (3)	0.6399 (2)	0.65605 (19)	0.0501 (6)	
O1	0.6771 (4)	1.2526 (3)	0.9840 (2)	0.1026 (11)	
O2	0.9903 (3)	1.1379 (3)	0.7756 (2)	0.0987 (10)	
O3	0.3260 (3)	0.7192 (2)	0.42331 (18)	0.0797 (8)	
O4	0.2088 (3)	0.7188 (2)	0.5685 (2)	0.0766 (8)	
C2	0.7994 (4)	1.2237 (3)	0.9760 (3)	0.0626 (9)	
C3	0.9105 (4)	1.2127 (3)	1.0630 (2)	0.0547 (8)	
C4	0.8859 (5)	1.2441 (4)	1.1612 (3)	0.0767 (11)	
H4	0.7994	1.2747	1.1724	0.092*	
C5	0.9881 (6)	1.2309 (4)	1.2432 (3)	0.0864 (13)	
H5	0.9706	1.2547	1.3092	0.104*	
C6	1.1117 (6)	1.1846 (4)	1.2296 (3)	0.0803 (13)	
H6	1.1779	1.1753	1.2858	0.096*	
C7	1.1429 (4)	1.1495 (3)	1.1304 (3)	0.0645 (10)	
C8	1.2697 (5)	1.1010 (4)	1.1103 (5)	0.0918 (15)	
H8	1.3383	1.0895	1.1643	0.110*	
C9	1.2953 (5)	1.0705 (4)	1.0149 (6)	0.1053 (19)	
H9	1.3799	1.0370	1.0038	0.126*	
C10	1.1970 (5)	1.0884 (4)	0.9325 (4)	0.0845 (13)	
H10	1.2170	1.0683	0.8672	0.101*	
C11	1.0701 (4)	1.1359 (3)	0.9479 (3)	0.0547 (8)	
C12	1.0410 (4)	1.1663 (3)	1.0464 (3)	0.0492 (8)	
C13	0.9662 (4)	1.1572 (3)	0.8612 (3)	0.0636 (10)	
C14	0.7269 (5)	1.2149 (3)	0.7930 (3)	0.0737 (11)	
H14A	0.7827	1.2462	0.7515	0.088*	
H14B	0.6660	1.2723	0.8202	0.088*	
C15	0.6233 (4)	1.0984 (3)	0.7248 (3)	0.0602 (9)	
C16	0.6623 (5)	1.0275 (3)	0.6388 (3)	0.0680 (10)	
H16	0.7496	1.0539	0.6214	0.082*	
C17	0.5738 (4)	0.9184 (3)	0.5784 (3)	0.0636 (9)	
H17	0.6009	0.8715	0.5208	0.076*	
C18	0.4443 (4)	0.8797 (3)	0.6049 (2)	0.0535 (8)	
C19	0.4011 (4)	0.9501 (4)	0.6880 (3)	0.0714 (10)	
H19	0.3127	0.9246	0.7045	0.086*	
C20	0.4909 (5)	1.0596 (4)	0.7469 (3)	0.0765 (11)	
H20	0.4611	1.1079	0.8027	0.092*	
C21	0.5844 (4)	0.5873 (3)	0.6806 (2)	0.0500 (8)	
H21	0.6372	0.5541	0.6320	0.060*	
C22	0.6255 (4)	0.5781 (3)	0.7849 (2)	0.0475 (7)	
C23	0.7517 (4)	0.5304 (3)	0.8140 (3)	0.0580 (9)	
H23	0.8057	0.4994	0.7655	0.070*	
C24	0.7984 (4)	0.5285 (3)	0.9143 (3)	0.0613 (9)	
H24	0.8839	0.4973	0.9341	0.074*	
C25	0.7162 (4)	0.5734 (3)	0.9838 (2)	0.0550 (8)	
C26	0.5894 (4)	0.6196 (3)	0.9578 (3)	0.0638 (9)	
H26	0.5356	0.6495	1.0067	0.077*	
C27	0.5431 (4)	0.6209 (3)	0.8576 (3)	0.0615 (9)	
H27	0.4561	0.6507	0.8382	0.074*	
N4	0.7691 (4)	0.5767 (3)	1.0923 (3)	0.0733 (9)	
O5	0.6974 (4)	0.6209 (4)	1.1535 (2)	0.1111 (12)	
O6	0.8819 (4)	0.5387 (4)	1.1166 (2)	0.1188 (13)	
S2	0.74498 (12)	0.64956 (10)	0.39415 (8)	0.0747 (3)	
O1S	0.7208 (3)	0.6673 (3)	0.5003 (2)	0.0830 (8)	
C1S	0.8748 (5)	0.5522 (4)	0.3777 (3)	0.0833 (12)	
H1S1	0.8257	0.4734	0.3689	0.125*	
H1S2	0.9591	0.5784	0.4379	0.125*	
H1S3	0.9105	0.5515	0.3173	0.125*	
C2S	0.8693 (6)	0.7814 (4)	0.4070 (4)	0.1081 (17)	
H2S1	0.8157	0.8468	0.4170	0.162*	
H2S2	0.9049	0.7705	0.3451	0.162*	
H2S3	0.9542	0.7979	0.4657	0.162*	

### Atomic displacement parameters (Å^2^)

**Table:**

	*U*^11^	*U*^22^	*U*^33^	*U*^12^	*U*^13^	*U*^23^
S1	0.0667 (6)	0.0595 (5)	0.0449 (5)	−0.0013 (4)	−0.0036 (4)	0.0219 (4)
N1	0.0665 (19)	0.0523 (16)	0.0478 (16)	0.0087 (14)	0.0064 (14)	0.0155 (13)
N2	0.0696 (19)	0.0552 (16)	0.0371 (14)	0.0016 (14)	0.0122 (14)	0.0193 (12)
N3	0.0566 (16)	0.0536 (15)	0.0412 (14)	0.0033 (13)	0.0094 (12)	0.0217 (12)
O1	0.084 (2)	0.137 (3)	0.079 (2)	0.060 (2)	0.0151 (16)	0.0140 (19)
O2	0.103 (2)	0.127 (3)	0.0623 (18)	−0.0099 (19)	0.0389 (17)	0.0240 (17)
O3	0.113 (2)	0.0723 (17)	0.0391 (13)	−0.0025 (15)	−0.0086 (13)	0.0230 (12)
O4	0.0539 (15)	0.0878 (19)	0.0830 (19)	0.0021 (13)	0.0062 (13)	0.0319 (15)
C2	0.064 (2)	0.062 (2)	0.055 (2)	0.0181 (18)	0.0125 (18)	0.0088 (17)
C3	0.059 (2)	0.055 (2)	0.0449 (19)	0.0089 (16)	0.0094 (16)	0.0111 (15)
C4	0.077 (3)	0.085 (3)	0.059 (2)	0.014 (2)	0.019 (2)	0.010 (2)
C5	0.103 (4)	0.097 (3)	0.046 (2)	−0.003 (3)	0.011 (2)	0.019 (2)
C6	0.094 (3)	0.068 (3)	0.062 (3)	−0.011 (2)	−0.012 (2)	0.028 (2)
C7	0.058 (2)	0.0424 (18)	0.079 (3)	−0.0036 (16)	−0.0068 (19)	0.0200 (17)
C8	0.062 (3)	0.057 (2)	0.136 (5)	0.007 (2)	−0.012 (3)	0.028 (3)
C9	0.053 (3)	0.075 (3)	0.170 (6)	0.020 (2)	0.022 (3)	0.015 (3)
C10	0.062 (3)	0.068 (3)	0.111 (4)	0.003 (2)	0.038 (3)	0.004 (2)
C11	0.0454 (19)	0.0446 (17)	0.066 (2)	−0.0026 (15)	0.0159 (17)	0.0083 (16)
C12	0.0477 (19)	0.0370 (16)	0.057 (2)	−0.0010 (14)	0.0091 (15)	0.0119 (14)
C13	0.073 (3)	0.054 (2)	0.058 (2)	−0.0128 (18)	0.0226 (19)	0.0129 (17)
C14	0.099 (3)	0.054 (2)	0.058 (2)	0.008 (2)	−0.003 (2)	0.0214 (17)
C15	0.083 (3)	0.0477 (19)	0.0437 (19)	0.0127 (18)	−0.0020 (18)	0.0186 (15)
C16	0.082 (3)	0.060 (2)	0.059 (2)	−0.0055 (19)	0.014 (2)	0.0247 (18)
C17	0.080 (3)	0.056 (2)	0.051 (2)	0.0032 (19)	0.0152 (19)	0.0167 (16)
C18	0.062 (2)	0.0540 (19)	0.0415 (18)	0.0084 (16)	0.0002 (15)	0.0211 (15)
C19	0.067 (2)	0.080 (3)	0.061 (2)	0.007 (2)	0.0146 (19)	0.017 (2)
C20	0.080 (3)	0.072 (3)	0.060 (2)	0.009 (2)	0.012 (2)	0.004 (2)
C21	0.061 (2)	0.0471 (17)	0.0396 (17)	0.0028 (16)	0.0122 (15)	0.0141 (14)
C22	0.0561 (19)	0.0409 (16)	0.0446 (17)	0.0047 (14)	0.0108 (15)	0.0152 (13)
C23	0.073 (2)	0.053 (2)	0.051 (2)	0.0212 (17)	0.0210 (17)	0.0152 (16)
C24	0.069 (2)	0.062 (2)	0.055 (2)	0.0231 (18)	0.0075 (18)	0.0227 (17)
C25	0.064 (2)	0.055 (2)	0.0431 (18)	0.0007 (17)	0.0074 (16)	0.0192 (15)
C26	0.068 (2)	0.081 (3)	0.053 (2)	0.017 (2)	0.0235 (18)	0.0292 (19)
C27	0.057 (2)	0.083 (3)	0.057 (2)	0.0212 (19)	0.0184 (17)	0.0352 (19)
N4	0.080 (2)	0.087 (2)	0.052 (2)	0.0088 (19)	0.0094 (18)	0.0279 (17)
O5	0.115 (3)	0.171 (3)	0.0557 (18)	0.032 (2)	0.0289 (19)	0.043 (2)
O6	0.121 (3)	0.182 (4)	0.072 (2)	0.070 (3)	0.0121 (19)	0.062 (2)
S2	0.0732 (7)	0.1007 (8)	0.0565 (6)	0.0068 (6)	0.0234 (5)	0.0332 (5)
O1S	0.103 (2)	0.095 (2)	0.0608 (16)	0.0099 (16)	0.0414 (15)	0.0291 (14)
C1S	0.080 (3)	0.094 (3)	0.077 (3)	0.010 (2)	0.028 (2)	0.025 (2)
C2S	0.130 (4)	0.101 (4)	0.115 (4)	0.005 (3)	0.060 (3)	0.053 (3)

### Geometric parameters (Å, °)

**Table:**

S1—O4	1.423 (3)	C15—C20	1.375 (5)
S1—O3	1.429 (2)	C15—C16	1.384 (5)
S1—N2	1.630 (3)	C16—C17	1.381 (5)
S1—C18	1.762 (3)	C16—H16	0.9300
N1—C2	1.388 (4)	C17—C18	1.383 (5)
N1—C13	1.394 (5)	C17—H17	0.9300
N1—C14	1.478 (4)	C18—C19	1.373 (5)
N2—N3	1.394 (3)	C19—C20	1.383 (5)
N2—H2N	0.8006	C19—H19	0.9300
N3—C21	1.263 (4)	C20—H20	0.9300
O1—C2	1.224 (4)	C21—C22	1.462 (4)
O2—C13	1.214 (4)	C21—H21	0.9300
C2—C3	1.461 (5)	C22—C23	1.383 (4)
C3—C4	1.377 (5)	C22—C27	1.394 (4)
C3—C12	1.404 (5)	C23—C24	1.381 (5)
C4—C5	1.380 (6)	C23—H23	0.9300
C4—H4	0.9300	C24—C25	1.367 (5)
C5—C6	1.336 (6)	C24—H24	0.9300
C5—H5	0.9300	C25—C26	1.369 (5)
C6—C7	1.415 (6)	C25—N4	1.474 (4)
C6—H6	0.9300	C26—C27	1.377 (5)
C7—C8	1.395 (6)	C26—H26	0.9300
C7—C12	1.418 (5)	C27—H27	0.9300
C8—C9	1.344 (7)	N4—O6	1.200 (4)
C8—H8	0.9300	N4—O5	1.213 (4)
C9—C10	1.391 (7)	S2—O1S	1.494 (3)
C9—H9	0.9300	S2—C1S	1.755 (4)
C10—C11	1.375 (5)	S2—C2S	1.781 (5)
C10—H10	0.9300	C1S—H1S1	0.9600
C11—C12	1.399 (5)	C1S—H1S2	0.9600
C11—C13	1.481 (5)	C1S—H1S3	0.9600
C14—C15	1.517 (5)	C2S—H2S1	0.9600
C14—H14A	0.9700	C2S—H2S2	0.9600
C14—H14B	0.9700	C2S—H2S3	0.9600
			
O4—S1—O3	120.25 (16)	C20—C15—C14	121.5 (4)
O4—S1—N2	108.61 (16)	C16—C15—C14	120.0 (4)
O3—S1—N2	103.64 (16)	C17—C16—C15	121.1 (4)
O4—S1—C18	107.94 (17)	C17—C16—H16	119.5
O3—S1—C18	109.03 (15)	C15—C16—H16	119.5
N2—S1—C18	106.58 (15)	C16—C17—C18	119.1 (4)
C2—N1—C13	123.8 (3)	C16—C17—H17	120.4
C2—N1—C14	118.4 (3)	C18—C17—H17	120.4
C13—N1—C14	117.7 (3)	C19—C18—C17	120.7 (3)
N3—N2—S1	115.3 (2)	C19—C18—S1	121.3 (3)
N3—N2—H2N	117.1	C17—C18—S1	118.0 (3)
S1—N2—H2N	114.1	C18—C19—C20	119.2 (4)
C21—N3—N2	115.5 (3)	C18—C19—H19	120.4
O1—C2—N1	119.3 (3)	C20—C19—H19	120.4
O1—C2—C3	122.8 (3)	C15—C20—C19	121.4 (4)
N1—C2—C3	117.9 (3)	C15—C20—H20	119.3
C4—C3—C12	119.4 (3)	C19—C20—H20	119.3
C4—C3—C2	120.4 (3)	N3—C21—C22	120.5 (3)
C12—C3—C2	120.1 (3)	N3—C21—H21	119.7
C3—C4—C5	120.8 (4)	C22—C21—H21	119.7
C3—C4—H4	119.6	C23—C22—C27	118.9 (3)
C5—C4—H4	119.6	C23—C22—C21	119.9 (3)
C6—C5—C4	121.3 (4)	C27—C22—C21	121.1 (3)
C6—C5—H5	119.4	C24—C23—C22	120.7 (3)
C4—C5—H5	119.4	C24—C23—H23	119.6
C5—C6—C7	120.7 (4)	C22—C23—H23	119.6
C5—C6—H6	119.6	C25—C24—C23	118.5 (3)
C7—C6—H6	119.6	C25—C24—H24	120.7
C8—C7—C6	124.0 (4)	C23—C24—H24	120.7
C8—C7—C12	117.6 (4)	C24—C25—C26	122.6 (3)
C6—C7—C12	118.3 (4)	C24—C25—N4	118.9 (3)
C9—C8—C7	121.7 (5)	C26—C25—N4	118.5 (3)
C9—C8—H8	119.1	C25—C26—C27	118.6 (3)
C7—C8—H8	119.1	C25—C26—H26	120.7
C8—C9—C10	120.9 (4)	C27—C26—H26	120.7
C8—C9—H9	119.6	C26—C27—C22	120.6 (3)
C10—C9—H9	119.6	C26—C27—H27	119.7
C11—C10—C9	119.9 (5)	C22—C27—H27	119.7
C11—C10—H10	120.1	O6—N4—O5	123.1 (4)
C9—C10—H10	120.1	O6—N4—C25	119.1 (4)
C10—C11—C12	119.8 (4)	O5—N4—C25	117.7 (4)
C10—C11—C13	120.2 (4)	O1S—S2—C1S	106.61 (19)
C12—C11—C13	119.9 (3)	O1S—S2—C2S	104.8 (2)
C11—C12—C3	120.5 (3)	C1S—S2—C2S	97.5 (2)
C11—C12—C7	120.1 (3)	S2—C1S—H1S1	109.5
C3—C12—C7	119.4 (3)	S2—C1S—H1S2	109.5
O2—C13—N1	120.1 (4)	H1S1—C1S—H1S2	109.5
O2—C13—C11	122.6 (4)	S2—C1S—H1S3	109.5
N1—C13—C11	117.3 (3)	H1S1—C1S—H1S3	109.5
N1—C14—C15	111.5 (3)	H1S2—C1S—H1S3	109.5
N1—C14—H14A	109.3	S2—C2S—H2S1	109.5
C15—C14—H14A	109.3	S2—C2S—H2S2	109.5
N1—C14—H14B	109.3	H2S1—C2S—H2S2	109.5
C15—C14—H14B	109.3	S2—C2S—H2S3	109.5
H14A—C14—H14B	108.0	H2S1—C2S—H2S3	109.5
C20—C15—C16	118.5 (3)	H2S2—C2S—H2S3	109.5
			
O4—S1—N2—N3	−49.9 (3)	C12—C11—C13—O2	176.2 (3)
O3—S1—N2—N3	−178.9 (2)	C10—C11—C13—N1	176.5 (3)
C18—S1—N2—N3	66.1 (3)	C12—C11—C13—N1	−4.8 (4)
S1—N2—N3—C21	−165.8 (2)	C2—N1—C14—C15	−96.1 (4)
C13—N1—C2—O1	−175.1 (3)	C13—N1—C14—C15	82.0 (4)
C14—N1—C2—O1	2.9 (5)	N1—C14—C15—C20	86.5 (5)
C13—N1—C2—C3	4.6 (5)	N1—C14—C15—C16	−92.5 (4)
C14—N1—C2—C3	−177.4 (3)	C20—C15—C16—C17	−2.3 (5)
O1—C2—C3—C4	−5.1 (6)	C14—C15—C16—C17	176.8 (3)
N1—C2—C3—C4	175.2 (3)	C15—C16—C17—C18	−0.1 (5)
O1—C2—C3—C12	172.8 (4)	C16—C17—C18—C19	2.1 (5)
N1—C2—C3—C12	−6.8 (5)	C16—C17—C18—S1	−176.1 (3)
C12—C3—C4—C5	0.2 (6)	O4—S1—C18—C19	5.1 (3)
C2—C3—C4—C5	178.1 (4)	O3—S1—C18—C19	137.3 (3)
C3—C4—C5—C6	−1.7 (7)	N2—S1—C18—C19	−111.5 (3)
C4—C5—C6—C7	1.1 (7)	O4—S1—C18—C17	−176.8 (3)
C5—C6—C7—C8	179.7 (4)	O3—S1—C18—C17	−44.6 (3)
C5—C6—C7—C12	0.8 (5)	N2—S1—C18—C17	66.7 (3)
C6—C7—C8—C9	−179.1 (4)	C17—C18—C19—C20	−1.6 (5)
C12—C7—C8—C9	−0.2 (6)	S1—C18—C19—C20	176.6 (3)
C7—C8—C9—C10	1.2 (7)	C16—C15—C20—C19	2.8 (6)
C8—C9—C10—C11	−1.2 (7)	C14—C15—C20—C19	−176.2 (3)
C9—C10—C11—C12	0.2 (5)	C18—C19—C20—C15	−0.9 (6)
C9—C10—C11—C13	179.0 (4)	N2—N3—C21—C22	−179.6 (2)
C10—C11—C12—C3	−178.8 (3)	N3—C21—C22—C23	−173.8 (3)
C13—C11—C12—C3	2.5 (4)	N3—C21—C22—C27	3.6 (5)
C10—C11—C12—C7	0.7 (5)	C27—C22—C23—C24	−2.0 (5)
C13—C11—C12—C7	−178.0 (3)	C21—C22—C23—C24	175.5 (3)
C4—C3—C12—C11	−178.7 (3)	C22—C23—C24—C25	0.7 (5)
C2—C3—C12—C11	3.3 (5)	C23—C24—C25—C26	0.3 (5)
C4—C3—C12—C7	1.8 (5)	C23—C24—C25—N4	−177.2 (3)
C2—C3—C12—C7	−176.2 (3)	C24—C25—C26—C27	−0.1 (5)
C8—C7—C12—C11	−0.7 (5)	N4—C25—C26—C27	177.5 (3)
C6—C7—C12—C11	178.2 (3)	C25—C26—C27—C22	−1.2 (5)
C8—C7—C12—C3	178.8 (3)	C23—C22—C27—C26	2.2 (5)
C6—C7—C12—C3	−2.3 (5)	C21—C22—C27—C26	−175.3 (3)
C2—N1—C13—O2	−179.9 (3)	C24—C25—N4—O6	−0.8 (5)
C14—N1—C13—O2	2.1 (5)	C26—C25—N4—O6	−178.5 (4)
C2—N1—C13—C11	1.1 (5)	C24—C25—N4—O5	177.4 (4)
C14—N1—C13—C11	−176.9 (3)	C26—C25—N4—O5	−0.3 (5)
C10—C11—C13—O2	−2.5 (5)		

### Hydrogen-bond geometry (Å, °)

**Table:**

Cg is the centroid of the *p*-nitrophenyl (C22–C27) ring.

**Table:**

*D*—H···*A*	*D*—H	H···*A*	*D*···*A*	*D*—H···*A*
N2—H2N···O1S	0.80	1.99	2.764 (4)	163
C8—H8···Cg^i^	0.93	2.90	3.799 (6)	162

Symmetry codes: (i) −*x*+2, −*y*+2, −*z*+2.
